# Internalization of rabies virus glycoprotein differs between pathogenic and attenuated virus strains

**DOI:** 10.1099/jgv.0.001935

**Published:** 2023-12-08

**Authors:** Ibrahim Almasoud, Frank W. Charlton, Stefan Finke, John N. Barr, Jamel Mankouri

**Affiliations:** ^1^​ School of Molecular and Cellular Biology, Faculty of Biological Sciences, University of Leeds, Leeds LS2 9JT, UK; ^2^​ Institute of Molecular Virology and Cell Biology, Friedrich-Loeffler-Institut (FLI), Federal Research Institute for Animal Health, Greifswald-Insel Riems, Germany; ^†^​ Present address: Department of Biological Sciences, Faculty of Science, Kuwait University, Kuwait City, Kuwait

**Keywords:** attenuated vaccine strain SAD-B19, challenge virus standard (CVS) strain, internalization, trafficking, rabies virus, rabies virus glycoprotein

## Abstract

The zoonotic rabies virus (RABV) is a non-segmented negative-sense RNA virus classified within the family *Rhabdoviridae*, and is the most common aetiological agent responsible for fatal rabies disease. The RABV glycoprotein (G) forms trimeric spikes that protrude from RABV virions and mediate virus attachment, entry and spread, and is a major determinant of RABV pathogenesis. A range of RABV strains exist that are highly pathogenic in part due to their ability to evade host immune detection. However, some strains are disease-attenuated and can be cleared by host defences. A detailed molecular understanding of how strain variation relates to pathogenesis is currently lacking. Here, we reveal key differences in the trafficking profiles of RABV-G proteins from the challenge virus standard strain (CVS-11) and a highly attenuated vaccine strain SAD-B19 (SAD). We show that CVS-G traffics to the cell surface and undergoes rapid internalization through both clathrin- and cholesterol-dependent endocytic pathways. In contrast, SAD-G remains resident at the plasma membrane and internalizes at a significantly slower rate. Through engineering hybrids of CVS-G and SAD-G, we show that the cytoplasmic tail of CVS-G is the key determinant of these different internalization profiles. Alanine scanning further revealed that mutation of Y497 in CVS-G (H497 in SAD-G) could reduce the rate of internalization to SAD-G levels. Together, these data reveal new phenotypic differences between CVS-G and SAD-G proteins that may contribute to altered *in vivo* pathogenicity.

## Introduction

Rabies is a fatal zoonotic disease typified by a progressive encephalomyelitis in infected individuals [1] that is almost invariably fatal [2, 3]. According to the World Health Organization (WHO), rabies virus (RABV) is present in every continent on earth except Antarctica and afflicts approximately 150 territories worldwide [4]. To date, the majority of RABV infections (~95 %) occur in rural communities throughout Africa and Asia [3], causing an estimated 39 000–59 000 deaths each year. However, these numbers likely represent a gross underestimation due to inadequate virus surveillance in many afflicted regions. Furthermore, 30–50 % of infected individuals are children under the age of 15 years, meaning that RABV accounts for 1.74 million disability-adjusted life years (DALYs) lost each year [4].

RABV is a lyssavirus of the family *Rhabdoviridae*, enveloped, bullet-shaped negative-sense RNA viruses [5, 6]. RABV encodes five viral proteins from a ~12 kb genome, including an RNA-binding nucleoprotein (RABV-N), a phosphoprotein (RABV-P), a structural matrix protein (RABV-M), a viral RNA polymerase (RABV-L) and the surface-expressed glycoprotein (RABV-G) [5]. RABV is neuroinvasive and infects the central nervous system (CNS) from peripheral sites [5, 7].

A number of pathogenic or attenuated RABV strains exist, but the mechanism(s) governing the different outcomes of virus infection remain poorly defined. Despite their lethality, pathogenic RABV strains cause only mild levels of neuronal inflammation and low levels of cell death [8]. In contrast, attenuated RABV strains induce extensive inflammation [9], which can lead to their clearance by host defences [10]. Pathogenic RABV strains display pronounced astrocyte infectivity, whereas attenuated RABV strains show abortive astrocyte infection, triggering innate immunity in the CNS [11]. The pathogenicity of RABV has been largely attributed to type-I interferon antagonistic activities [10, 11] and RABV-G [12, 13]. It was previously reported that pathogenic RABV strains limit RABV-G expression to avoid immune surveillance, whilst attenuated strains overexpress RABV-G, which is more readily detected by host defences [14]. Consistent with these studies, engineered RABV strains that express an extra copy of RABV-G show lower lethality *in vivo* [15]. RABV-G retention in the endoplasmic reticulum occurs in highly virulent field RABV-infected cell cultures. Cell culture adaptation by mutations in the ectodomain correlates with increased RABV-G accumulation at the plasma membrane and more efficient infectious virus release caused by mutations in RABV-G ectodomain [16]. Exchanging RABV-G of the attenuated SAD-B19 (SAD) strain with RABV-G of the Challenge Virus Street (CVS) strain enhances pathogenicity [13, 17]. Although CVS viruses appear to be slightly attenuated due to P gene mutation affecting interferon antagonism [18], the glycoprotein G display many features of pathogenic RABV.

Here, we compared RABV-G proteins from CVS-11 and SAD-B19 to identify key characteristics that may contribute to this differential pathogenicity. We show that CVS-G is expressed at the cell surface and undergoes rapid internalization that reduces its surface expression, whilst SAD-G undergoes limited endocytosis. We further show that the internalization event occurs for other pathogenic RABV-G proteins and is mediated by several residues absent in SAD-B19, most notably Y497 in CVS-G (H497 in almost all attenuated strains of RABV).

## Results

### RABV-G from pathogenic virus strains undergo higher levels of internalization than SAD-G

**Fig. 1.**
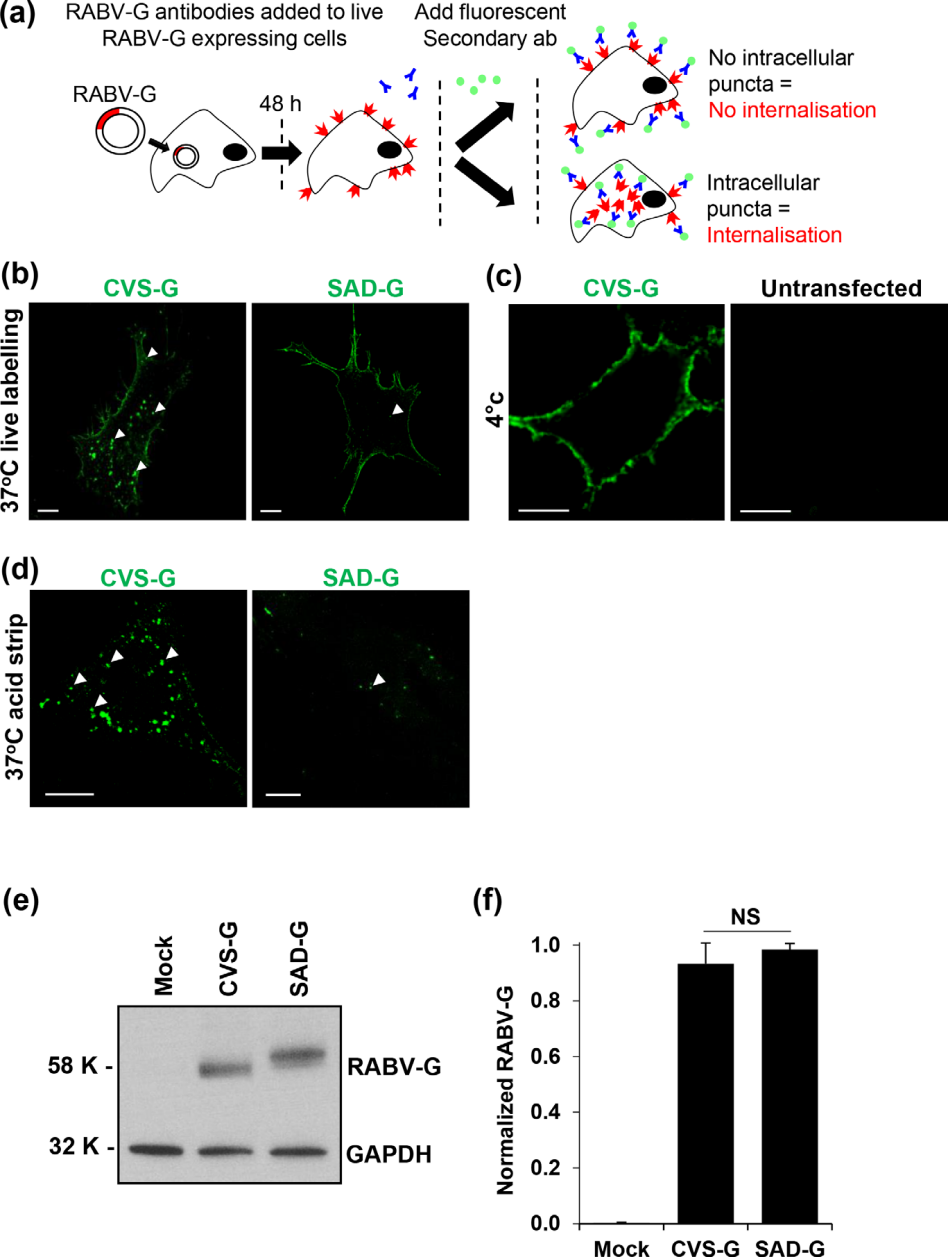
CVS-G undergoes higher levels of internalization than SAD-G. (**a**) Schematic representation of the RABV-G internalization assay. SVG-A cells were transfected with RABV-G and after 48 h anti-RABV-G antibodies were added to live unpermeabilized cells. Plasma membrane staining indicated surface-retained RABV-G, whereas presence of intracellular puncta indicated internalized RABV-G. (**b**) RABV-G expression in SVG-A cells shows a significant difference in intracellular puncta, with CVS-G exhibiting more intracellular puncta labelled from PM (white arrows) than SAD-G. Representative cell image shown out of >50 cell images. (**c**) Successful CVS-G labelling at 4 °C to visualize RABV-G expression at the PM, as compared to mock-transfected cells. SAD-G display similar phenotype to CVS-G (data not shown). (**d**) Acid stripping assay confirmed intracellular trafficking of RABV-Gs and that CVS-G has significantly more protected puncta than SAD-G (**e**) Western blot of mock, CVS-G- and SAD-G-expressing HEK293T cells shows equivalent overall expression levels, indicating that internalization differences are not synthesis-related (*n*=3). (**f**) Densitometry of *n*=3 Western blots in (**e**) normalized to each loading control. Error bars indicate mean±sem and significant difference determined using Student’s *t*-test, comparing SAD-G to CVS-G (ns, not significant, *P*=0.22).

To our knowledge, a detailed comparison of the internalization of attenuated and pathogenic RABV G proteins has not been performed to date. As RABV-G is resident at the plasma membrane (PM), we reasoned that it could be labelled at the cell surface of live cells, permitting a comparison of PM expression between pathogenic and attenuated G-proteins (Fig. 1a). CVS-G or SAD-G were therefore live labelled with primary antibodies against RABV-G and a specific Alexa 488 fluorophore for 90 min at 37* °*C in SVG-A astrocytes prior to fixation and imaging in unpermeablized cells. Upon analysis, CVS-G showed labelling at the plasma membrane and in distinct intracellular puncta (Fig. 1b), whilst the majority of SAD-G was cell surface retained. We reasoned that the puncta represented reinternalizing CVS RABV-G proteins as antibody labelling at 4 °C (a non-endocytic-permissive temperature) led to a loss of the intracellular puncta observed (Fig. 1c). The internalizing puncta of CVS-G were further confirmed as intracellular and not cell membrane-associated sub-cisternae as they were resistant to acid stripping, which successfully removed any cell-surface RABV-G antibody-labelled complexes (Fig. 1d). The differences between CVS-G and SAD-G proteins were not a result of variable expression confirmed through Western blot analysis (Fig. 1e, f). The apparent difference in band sizes is due to differences in glycosylation between CVS-G and SAD-G, as reported previously [19]. Unless otherwise stated, all immunofluorescence (IF) experiments were conducted in SVG-A astrocytes.

### Validating RABV-G internalization difference

**Fig. 2.**
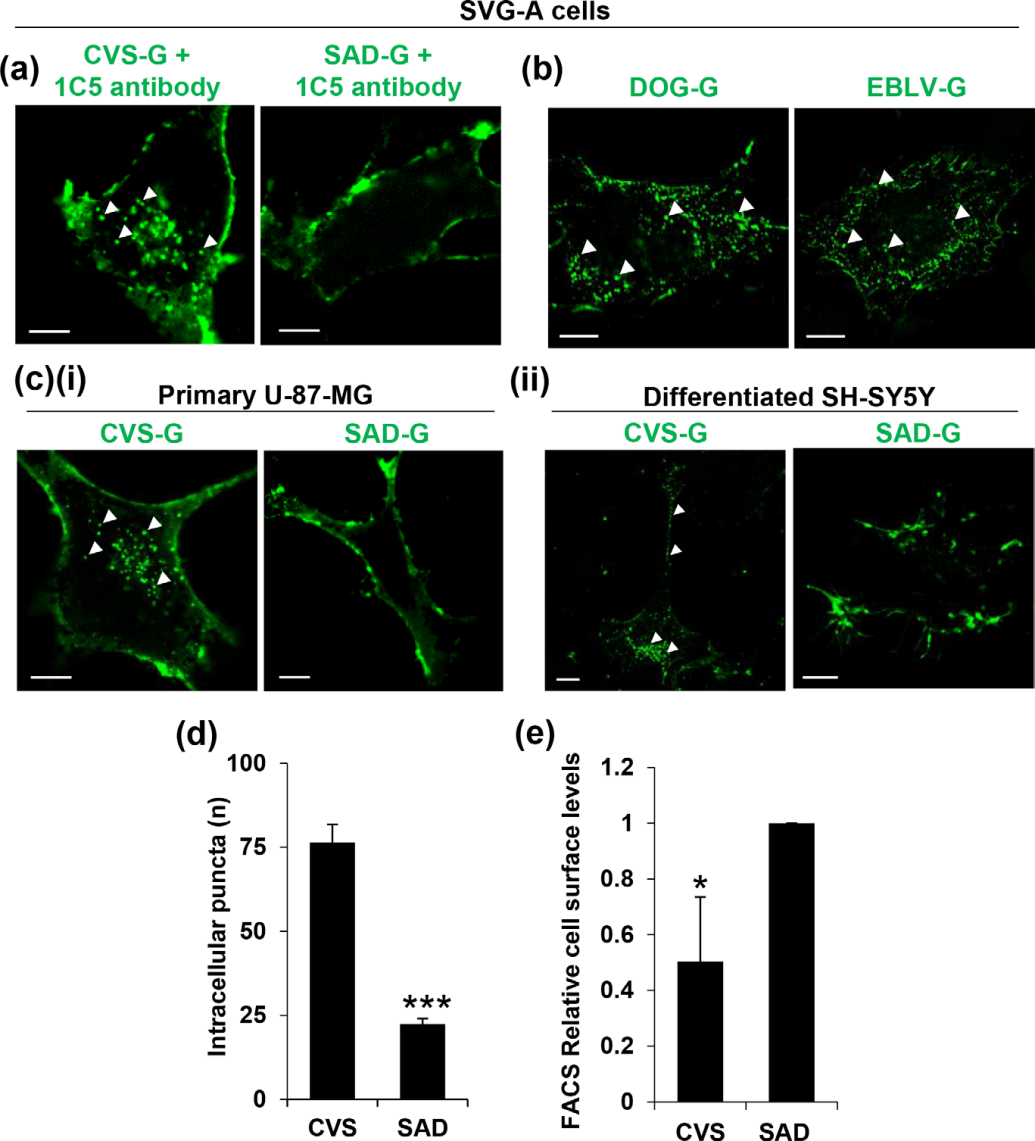
Validation and quantification of different internalization kinetics. (**a**) CVS-G and SAD-G display the same expression profile when stained using anti-G antibody (1C5) as seen with E559 anti-G. (**b**) Intracellular expression profile between CVS-G and other pathogenic strains such as DOG-G and EBLV-G is conserved, suggesting that internalization may relate to pathogenesis. (**c**) CVS-G and SAD-G display the same expression profile in (**i**) primary glial cells (U-87-MG) and (ii) differentiated neuronal-like SH-SY5Y cells. (**d**) Quantification of intracellular puncta, performed by counting internalized puncta in 50 different SVG-A cells, revealed a significant difference in puncta abundance between CVS-G and SAD-G. (**e**) SAD-G has higher PM expression levels, as seen through FACS flow cytometry analysis in SVG-A cells. Error bars indicate mean±sem. *, significant different between two conditions (*P*<0.05); ***, significant difference between two conditions (*P*<0.0005).

The IF assays shown above (Fig. 1b, d) used anti-RABV-G monoclonal antibody E559 (see Methods). To rule out the possibility of specific antibody-induced internalization, a distinct anti-RABV-G (1C5) was also used, which displayed the same internalization phenotype seen with E559 (Fig. 2a). We confirmed that this enhanced internalization phenotype was displayed by G-proteins from other pathogenic strains, as RABV-G from the street strain DOG (isolated in Azerbaijan) and the European bat 2 strain (EBLV) were also predominantly observed as internalized puncta (Fig. 2b). This internalization phenotype difference was also observed for glial derived U-87-MG cells and differentiated SH-SY5Y neuroblastoma cells (Fig. 2c). Upon quantification in SVG-A cells, CVS-G had an average of ~75 visible internalized puncta per cell, compared to ~20 internalized puncta for SAD-G under identical labelling conditions (Fig. 2d). Using FACS to quantify the surface levels of the RABV-G proteins, we also observed higher levels of PM expression for SAD-G compared to CVS-G, consistent with the notion that lower levels of SAD-G internalization leads to higher surface expression (Fig. 2e).

### Rabies G internalization time course alongside epidermal growth factor (EGF)

**Fig. 3.**
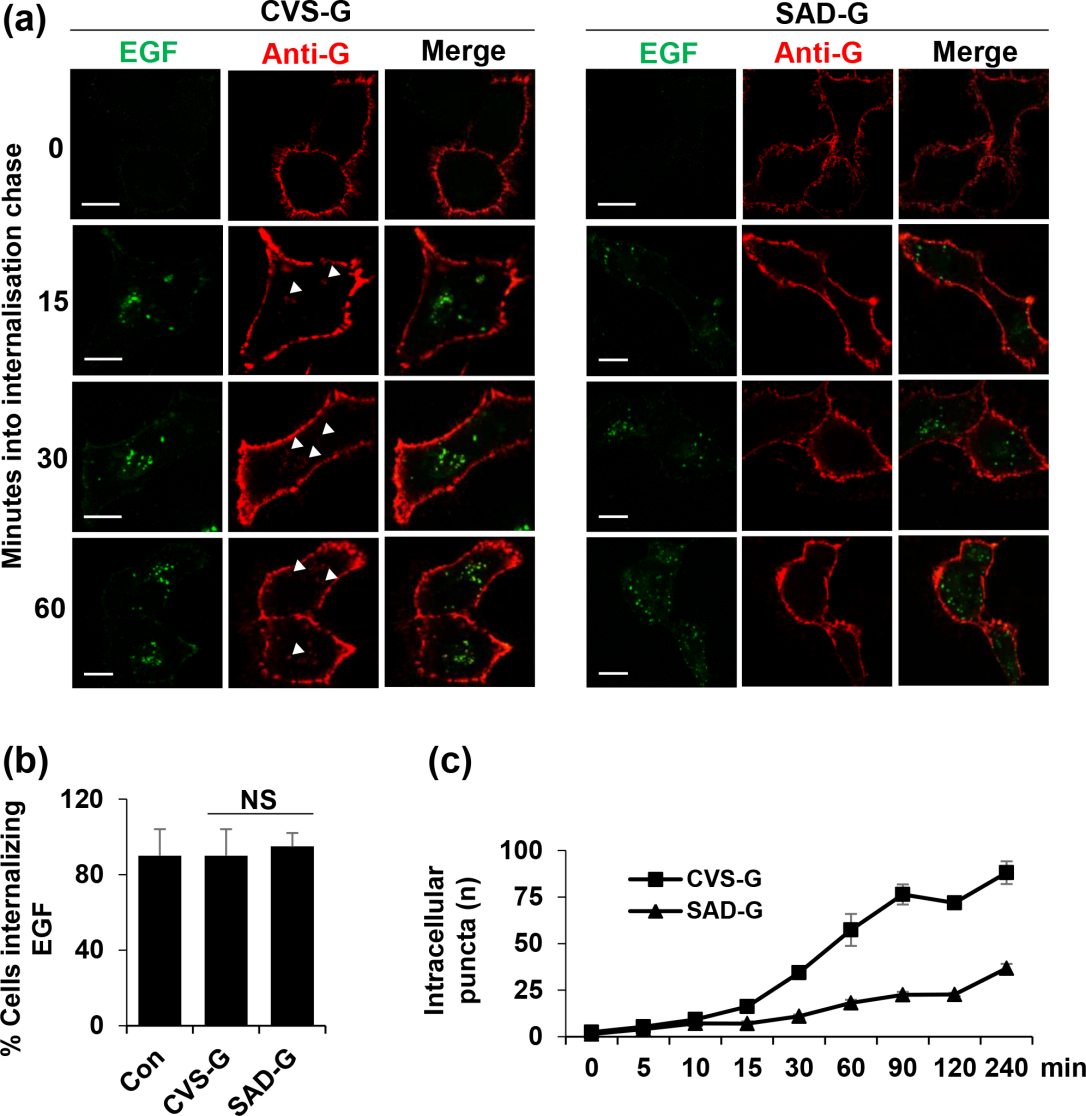
RABV-G internalization time course alongside EGF. (**a**) Selected time points (15, 30 and 60 min) showing that CVS-G has more internalizing puncta than SAD-G. RABV-G puncta internalized within 15 min (white arrows), almost as fast as EGF, whereas SAD-G showed almost no internalized puncta (**b**) Quantification of the number of cells internalizing EGF in mock-transfected cells versus cells expressing RABV-G show no significant difference between these cell populations. (**c**) Quantifications of each time point (25 cell images) show that internalization kinetics is time-dependent and CVS-G internalization is significantly higher than SAD-G after 15 min.

To rule out the possibility that SAD-G expression impairs general cellular endocytic processes, we co-labelled CVS-G- and SAD-G-expressing SVG-A cells with labelled EGF and assessed their co-uptake in live cells. Upon binding to EGFR, EGF undergoes endocytosis into late endosome/lysosomal compartments [20]. We observed comparable levels of internalization of EGF in mock-transfected, CVS-G- and SAD-G-expressing cells (Fig. 3a, b). This indicated that the reduced levels of intracellular SAD-G puncta were not a result of impaired cellular uptake processes.

We next investigated whether the phenotypic differences between the internalization of CVS-G and SAD-G were as a result of rate-specific cell surface removal by counting intracellular puncta over a 4 h time course (imaging started immediately after the addition of labelling antibodies). CVS-G was found to internalize within 15 min, at which time SAD-G remained almost exclusively PM associated (Fig. 3c, CVS-G puncta=16±2 vs SAD-G puncta 7±1). From 30 to 60 min, SAD-G did internalize but to lower levels that CVS-G (Fig. 3c, 30 min CVS-G puncta=35±3 SAD-G puncta=10±1, 60 min CVS-G puncta=58±9 SAD-G puncta=18±2), suggesting that the higher levels of CVS-G internal puncta were a result of its more rapid endocytosis.

Together, these data reveal a distinct trafficking phenotype that differs between pathogenic and attenuated RABV-G proteins, whereby pathogenic RABV-G is more rapidly endocytosed from the cell surface, leading to lower levels of PM expression.

### CVS-G internalizes through both clathrin- and caveolae-mediated endocytosis

**Fig. 4.**
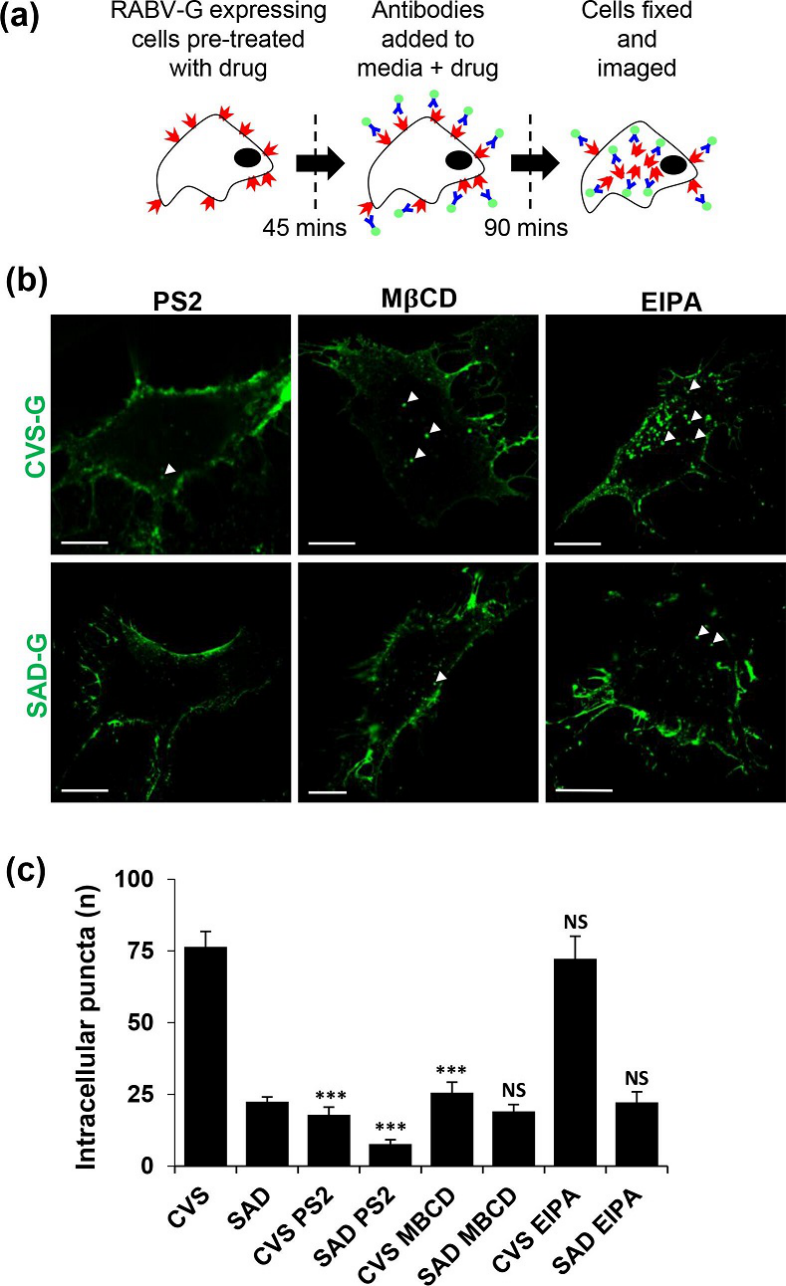
CVS-G internalization is mostly through CME and caveolae. (**a**) RABV-G-expressing cells were pre-treated with the drug for 45 min followed by 90 min treatment with antibodies+drugs. Then cells were fixed and imaged. (**b**) Representative images of each treatment. (**c**) Quantification of average number of intracellular puncta suggests that both RABV-G are affected by the CME inhibitor PitStop2 and only CVS-G is affected by the cholesterol-sequestering drug MβCD and neither RABV-G is affected by the micropinocytosis inhibitor EIPA. This suggests that both RABV-Gs are internalizing through CME and only CVS-G simultaneously use cholesterol-based entry that could be caveolae-based internalization.

We next assessed the route through which CVS-G internalizes. In the experiments that followed, SVG-A cells were pre-treated with characterized inhibitors of clathrin-, caveolae- and micropinocytosis-dependent pathways and RABV-G labelling was performed in the presence of each drug for 90 min (Fig. 4a, b). Following quantification (Fig. 4c), the CME inhibitor PitStop2 (PS) significantly inhibited ≥70 % of CVS-G internalization, confirming it as a major route of CVS-G endocytosis. Of interest, the SAD-G that did internalize was also inhibited by PS. We also observed a significant reduction of CVS-G internalization upon addition of the cholesterol-sequestering drug MβCD, which had no effect on SAD-G. EIPA, an inhibitor of macropinocytosis, did not influence either CVS-G or SAD-G. Together, these data suggest that CVS-G undergoes internalization in cells via both clathrin- and cholesterol-based internalization pathways.

### Role of the pathogenic RABV-G C-terminus in dictating its internalization phenotype

**Fig. 5.**
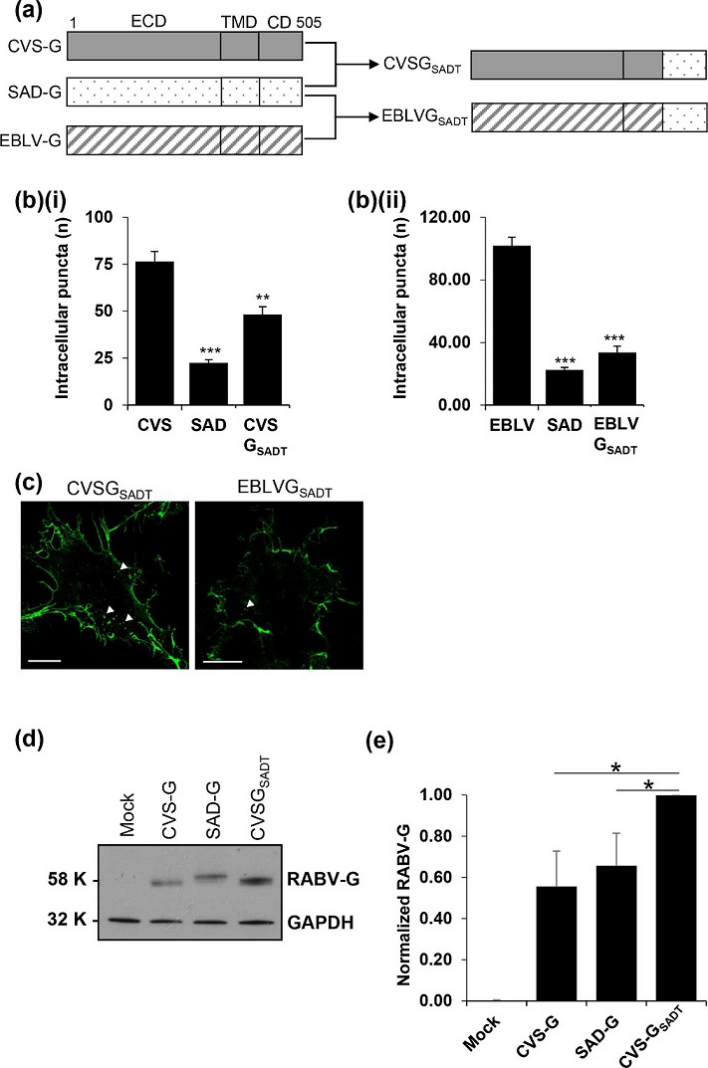
Internalization phenotype is partially dictated by the RABV-G C-terminus. (**a**) Schematics of the hybrid RABV-G constructs. The SAD-G tail domain replaced the tail domains of both CVS-G and EBLV-G, creating constructs CVSG_SADT_ and EBLVG_SADT_. (**b**) Quantifications of intracellular puncta suggest that (**i**) CVSG_SADT_ exhibits an intermediate internalization phenotype, whereas the phenotype of (ii) EBLVG_SADT_ closely represents that of SAD-G. (**c**) Representative images of internalization assay expression of CVSG_SADT_ and EBLVG_SADT_ corroborate the quantitative data. (**d**) Western blot of mock, CVS-G, SAD-G and CVSG_SADT_ show similar band sizes between CVS-G and CVSG_SADT_, although CVSG_SADT_ displays (**e**) higher expression levels.

We next investigated the regions of CVS-G that dictate its internalization, reasoning that its C-terminus would be exposed to cytosolic endocytosis regulators. Hybrid RABV-G proteins were created in which the short C-terminal cytoplasmic tail of pathogenic CVS-G or EBLV-G was replaced by the SAD-G cytoplasmic tail, termed CVSG_SADT_ or EBLVG_SADT_, respectively (Fig. 5a). In cells expressing these chimeric constructs, internalization was assessed following 90 min of live antibody labelling. We found that replacement of the CVS-G C-termini with SAD-G led to a~50 % reduction in its internalization, compared to an almost complete loss of EBLVG_SADT_ internalization (Fig. 5b, c), as compared to SAD-G-internalized puncta. Western blot analysis of CVS-G, SAD-G and CVSG_SADT_ shows similar expression pattern between CVS-G and CVSG_SADT_, although the latter displays higher expression levels (Fig. 5d, e). These data suggest that the C-terminal tails of both CVS-G and EBLV-G play a critical role in the internalization phenotype of both proteins.

### Identification of key residues in the C-terminus of CVS-G that influence its internalization behaviour

**Fig. 6.**
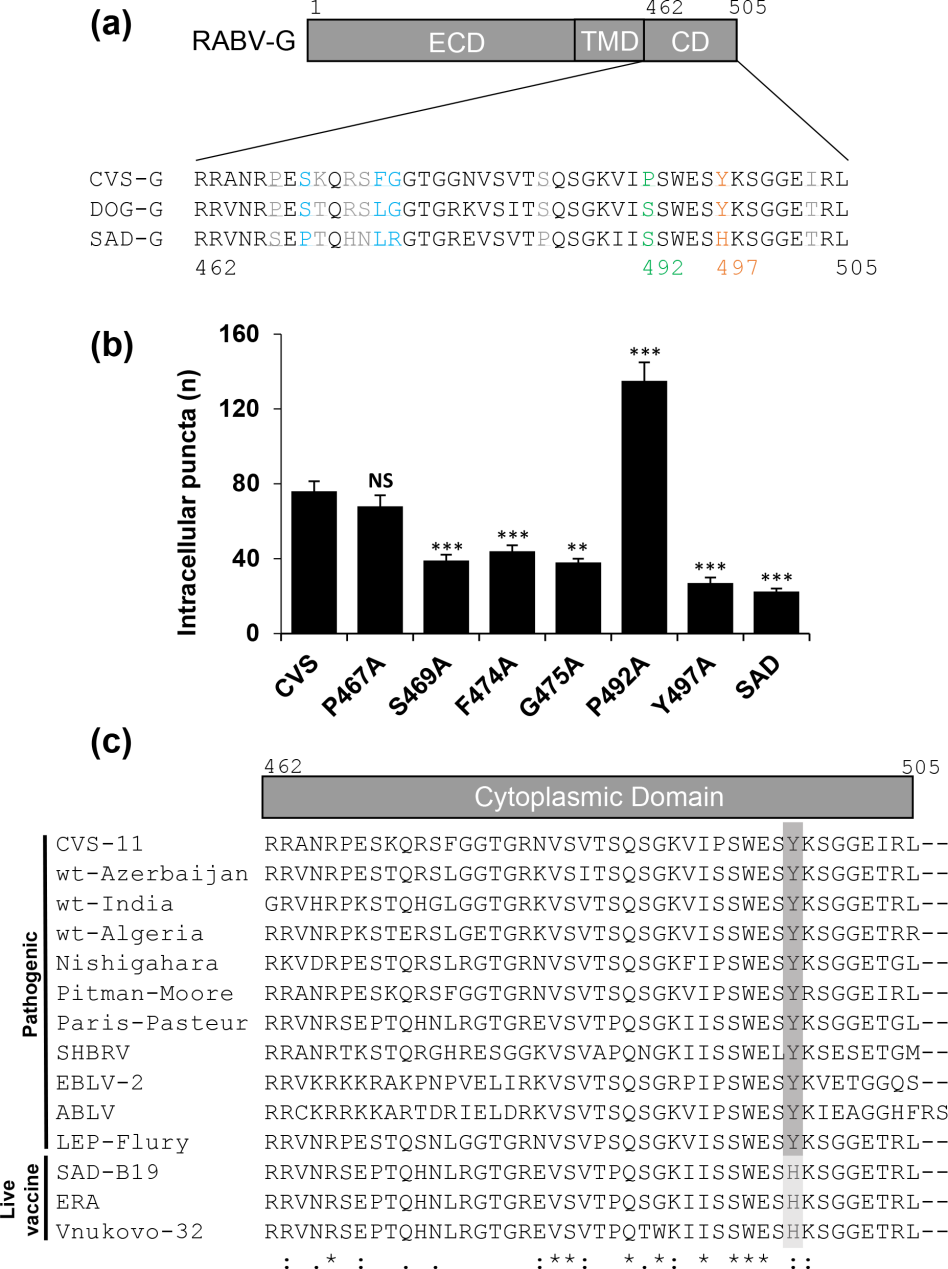
Residues in the C-terminus of CVS-G affect its internalization. (**a**) Schematic of the RABV-G structure with a zoom on its C-terminal tail. Eleven residues that are conserved between CVS-G and DOG-G but are different in SAD-G were mutated to alanine in CVS-G to probe their functional role. (**b**) Only five residues have an apparent effect on internalization, where four decreased internalization rates after being mutated to alanine and P_492_ significantly increased internalization rates. This proline substitution possibly destabilized the C-terminal structure, as prolines introduce kinks that are functionally important for host–RABV-G binding. (**c**) It seems that the only conserved mutation between pathogenic or SAD-derived live vaccine strains is Y or H at 497, respectively.

Following the observation that the short cytoplasmic tail of pathogenic RABV-G proteins influences their internalization, we performed alanine scanning of 11 residues that differ in the C-terminal tails of CVS-G and SAD-G, but do not differ between pathogenic DOG-G and CVS-G (Fig. 6a). Upon quantification of the average number of internalizing puncta of each CVS-G mutant, four residues (S_469_, F_474_, G_475_ and Y_497_) were identified as key to the internalization event, since their mutation to alanine led to a decreased number of internalizing puncta compared to wild-type CVS-G (Fig. 6b). Mutation of Y_497_ led to the most pronounced inhibition of CVS-G endocytosis, which is H_497_ in all attenuated vaccine strains based on SAD-B19 (Fig. 6c). Of interest, mutation of P_492_ significantly increased the number of internalizing CVS-G puncta, suggestive of more rapid surface removal [21, 22]. These data reveal key C-terminal residues in CVS-G that dictate its internalization behaviour.

## Discussion

This study is the first to demonstrate differences in the internalization of two viral RABV glycoproteins from pathogenic and attenuated strains. We show that internalization of CVS-G leads to reduced PM expression and occurs through both CME and cholesterol-associated endocytic pathways. We further show that the cytoplasmic domain of RABV-G is the major regulator of internalization and that a Y497H point mutation conserved between all SAD-B19-derived live vaccine strains impairs this endocytic event. These data reveal new mechanisms associated with RABV trafficking and the second reporting of a conserved point mutation in G significantly affecting RABV trafficking and potential pathogenesis, after R333Q [23].

### RABV-G glycosylation pattern

RABV G is N-glycosylated at N_37_ and N_319_ [24] and many tissue culture-adapted strains are subjected to additional glycosylation events at N_158_, N_204_ or N_247_, predicted to influence glycoprotein stability [25, 26]. Both CVS-G and SAD-G have the two conserved glycosylation sites at N_37_ and N_319_, but they differ in that CVS-G is further glycosylated at N_204_ and SAD-G is further glycosylated at N_247_. CVS_SADT_ displayed a similar Western blot band pattern to CVS-G in Fig. 5, confirming that the difference in Western blot band patterns is due to glycosylation, as both proteins share the same ectodomain and glycosylation pattern. CVS_SADT_ displays almost 50 % greater expression levels and this might be related to the amino acid sequence of this mutant protein.

### Cytoplasmic domain regulation of internalization

The hybrid glycoprotein EBLV_SADT_ displayed almost complete restoration of the SAD-G internalization phenotype, whereas CVS_SADT_ had partial restoration of the SAD-G phenotype. This potentially means that the sequence of the EBLV cytoplasmic domain has a greater effect on internalization than that of the CVS strain, and the internalization kinetics of CVS is regulated by other regions of RABV-G. The cytoplasmic domain of CVS is still, however, the major regulator of internalization. This observation is corroborated by previous work showing that a SHBRV-18 strain with SAD cytoplasmic tail (SHBRV_SADT_) exhibited partial restoration of SAD’s high plasma membrane expression [12] and, similar to CVS, its internalization kinetics is also regulated by other regions of RABV-G. The cytoplasmic domain is the most variable region of RABV-G and more comparative work on the sequence effect of RABV-G cytoplasmic domain might reveal a better characterized internalization motif.

### Cytoplasmic tyrosine-influenced internalization

Tyrosine residues in the cytoplasmic tails of proteins have long been associated with endocytic motifs [27–29]. The Y_497_ in RABV-G does not conform to a consensus YXXΦ motif, where X is any residue and Φ is a residue with a bulky side chain. RABV-G do not have such bulky Φ residues in the recognised position, seemingly ruling out involvement. However, Y_497_ sits within a conserved WXXYXXGG sequence, which could satisfy the requirements for an internalization motif in two ways – it is similar to the unconventional internalization motif YXXGL [30] or it might be a flipped YXXΦ sequence, as the upstream W is an amino acid with a bulky side chain. However, the contribution of identified upstream residues such as S_469_ and F_474_ in internalization enhancement hints at a more complex and non-canonical motif that is beyond the scope of this study to investigate.

All known SAD-derived live attenuated strains possess H_497_ instead of Y_497_ and we show that this point mutation is implicated in attenuation. Both SAD-B19 and Evelyn–Rokitnicky–Abelseth (ERA) were derived from the original pathogenic SAD strain through passaging in different sets of cell lines [31] and to see this conserved mutation between these attenuated strains hints at H_497_’s requirement for efficient replication in these mostly non-neuronal cell types. However, adaptation studies with a recombinant field virus RABV clone by limited cell culture passages on BHK cells passages reveals SAD-like mutations in the ectodomain, but not in the cytoplasmic domain [16]. It is conceivable that low-level RABV-G PM expression in pathogenic RABV strains is beneficial because of reduced immune recognition and clearance and thus longer time windows for virus replication and spread in the CNS.

Rabies is a progressive, fatal neurotropic virus and this finding might assist in the development of better designer vaccines, antivirals, or designer neurotracing tools with less cell cytotoxicity, the latter of which are based on mostly CVS-G and SAD-G [32, 33].

## Methods

### RABV-G expression constructs

Codon-optimized CVS-G and SAD-G were commercially synthesized (Invitrogen) and cloned into pcDNA3.1. Mutations were introduced via Q5 site-directed mutagenesis (New England Biolabs) and constructs were cloned into pCAGGS (Addgene). pCAGGS-CVS-G accession number is ABV24348.1 and pCAGGS-SAD-G accession number is P16288.1. pCAGGS-DOG-G (accession number CUI02214.1) and EBLV1-G (accession number SMD54588.1) have been described previously [16, 34]. pCAGGS-CVS_SADT_ and pCAGGS-EBLV_SADT_, comprising the SAD cytoplasmic tail sequence, were generated through PCR mutagenesis.

### Cell culture and transfection

SVG-A (human foetal astrocyte), U-87-MG (glia) and SH-SY5Y neuroblastoma cells were obtained from the European Collection of Cell Cultures (ECACC). Cells were cultured in Dulbecco’s modified Eagle’s medium (DMEM; Sigma) supplemented with 10 % foetal calf serum (FCS), 100 U ml^−1^ penicillin and 100 µg ml^−1^ streptomycin and maintained in a humidified incubator at 37 °C with 5 % CO_2_. SH-SY5Y cells were differentiated into neuronal-like cells according to previously described protocols [35]. All cells were transiently transfected with RABV-G constructs using Lipofectamine 2000 (Invitrogen) as per the manufacturer’s instructions.

### Antibodies and chemicals

Mouse monoclonal anti-RABV-G E559 and rabbit polyclonal anti-P serum P160 were described previously [34, 36]. Anti-RABV-G 1C5 antibodies were purchased from Abcam. GAPDH was obtained from Santa Cruz Biotechnology. PitStop2, MβCD and EIPA were purchased from Sigma-Aldrich. Alexa Fluor 488 and 594 anti-mouse antibodies were obtained from Molecular Probes. HRP-conjugated anti-mouse and HRP-conjugated anti-rabbit antibodies were purchased from Sigma-Aldrich.

### Western blot analysis

After transfection, HEK293T cells were lysed using a Leeds lysis buffer (LLB; 25 mM glycerol phosphate, 20 mM Tris, 150 mM NaCl, 1 mM EDTA, 1 % Triton X-100, 10 % glycerol, 50 mM NaF, 5 mM Na_4_O_7_P_2_, pH 7.4) with protease inhibitor cocktail (Invitrogen), for 15 min at 4 °C. Lysates were then collected, stored overnight and then run on 12 % sodium-dodecyl sulphate (SDS) gels using SDS-PAGE. Samples were then transferred onto PVDF membranes (Millipore) using a trans-blot turbo transfer system (Bio-Rad). PVDF membranes were then blocked in 10 % milk 0.1 % Tween in phosphate-buffered saline (PBS) for 1 h. Proteins were labelled with primary antibodies overnight at 4 °C and then with corresponding secondary antibodies for 1 h. Labelling was detected using the ECL chemiluminescence system and film exposed using an Xograph processor.

### Steady state immunofluorescence

SVG-A cells were plated onto sterilized poly-l-lysine (Sigma)-coated coverslips in 12-well plates (1×10^5^ cells per well) and allowed to adhere for 24 h. After transfection, cells were fixed with 4 % PFA for 10 min at 4 °C. Cells were then permeabilized with ice-cold 1 : 1 methanol : acetone solution for 10 min and blocked in 1 % bovine serum albumin (BSA; Sigma) in PBS for 15 min. Afterwards, cells were labelled with primary anti-RABV-G in 1 % BSA for 1 h, followed by three PBS washes and corresponding fluorescent Alexa-Flour 488 or 594 nm-conjugated antibodies for another 1 h and cells were washed four times in PBS. Cells were then labelled again with a different primary antibody and corresponding fluorescent Alexa-Fluor secondary antibody. After four PBS washings, cells were mounted using ProLong Gold anti-fade reagent with DAPI (Invitrogen) on glass slides.

### Internalization assay and FACS analyses

In 12- or 24-well plates and following transfection, live, unpermeabilized cells were labelled with anti-RABV-G and corresponding fluorescent Alexa-Fluor secondary in DMEM for 90 min (time chosen after time course signal noise optimization). After PBS washing, cells were not fixed and sent for fluorescence-activated cell sorting analysis (FACS) on a BD-LSR Fortessa (Becton Dickinson), using DiVa6 software.

### Surface stripping and cold labelling

After 90 min live labelling and before fixing cells, acidic glycine buffer (pH 2, 0.2 M glycine and 0.15 M NaCl) was added for 2 min to strip away cell surface antibody–glycoprotein complexes. Cold labelling was done by adding ice-cold primary–secondary antibody DMEM mix and leaving plates at 4 °C for 90 min until fixation.

### Image acquisition and analysis

An inverted laser scanning microscope (Zeiss LSM700) was used with an oil-immersion 40× objective lens for fluorescence imaging of fixed and labelled cells. The Fiji platform was used for data processing and image analysis. Internalizing puncta were quantified by counting the number of puncta per cell and averaging 50 cell images.
